# Signalling Overlaps between Nitrate and Auxin in Regulation of The Root System Architecture: Insights from the *Arabidopsis thaliana*

**DOI:** 10.3390/ijms21082880

**Published:** 2020-04-20

**Authors:** Muhammad Asim, Zia Ullah, Aluko Oluwaseun, Qian Wang, Haobao Liu

**Affiliations:** 1 Tobacco Research Institute, Chinese Academy of Agricultural Sciences, Qingdao 266101, China; asim.ktk91@aup.edu.pk (M.A.); zianust512@gmail.com (Z.U.);; 2 Graduate School of Chinese Academy of Agricultural Sciences, Beijing 100081, China

**Keywords:** nitrate, auxin, signalling crosstalk, nitrate responsive genes, root system architecture, *Arabidopsis*, primary root, lateral root

## Abstract

Nitrate (NO_3_^–^) and auxin are key regulators of root growth and development, modulating the signalling cascades in auxin-induced lateral root formation. Auxin biosynthesis, transport, and transduction are significantly altered by nitrate. A decrease in nitrate (NO_3_^–^) supply tends to promote auxin translocation from shoots to roots and vice-versa. This nitrate mediated auxin biosynthesis regulating lateral roots growth is induced by the nitrate transporters and its downstream transcription factors. Most nitrate responsive genes (short-term and long-term) are involved in signalling overlap between nitrate and auxin, thereby inducing lateral roots initiation, emergence, and development. Moreover, in the auxin signalling pathway, the varying nitrate supply regulates lateral roots development by modulating the auxin accumulation in the roots. Here, we focus on the roles of nitrate responsive genes in mediating auxin biosynthesis in *Arabidopsis* root, and the mechanism involved in the transport of auxin at different nitrate levels. In addition, this review also provides an insight into the significance of nitrate responsive regulatory module and their downstream transcription factors in root system architecture in the model plant *Arabidopsis thaliana*.

## 1. Introduction 

Roots are crucial for the anchorage of plants in the soil and facilitate the translocation of water and mineral nutrients. Thus, plant roots play an essential role in plant metabolism [1]. The root system is composed of primary root (embryonic roots) and postembryonic roots (lateral roots). Root system architecture (RSA) is the general spatial arrangement of individual parts of the root system and enables the plant to acquire adequate resources from the soil. The rate at which individual parts of the root system develop and grow can be reformed by their response to environmental signals, including changes in water, nutrient, and oxygen availability, or pathogens and pests [2,3].

Nitrogen (N) is in two major forms nitrate (NO_3_^−^) and ammonium (NH_4_^+^), which plays a vital role in the root growth and development of the plant. It was suggested that lateral roots (LRs) formation is regulated by NO_3_^−^ and NH_4_^+^ ratio via modification of the polar auxin transport [4]. NH_4_^+^ ions were found to dramatically suppress *Arabidopsis* root growth in the absence of K^+^, even though NO_3_^–^ is available [5]. The ammonia transporter gene *AtAmt1.1* has also been found to play a critical role in restructuring LRs architecture under N-starvation [6,7]. However, the majority of the plants preferentially uptake nitrogen in the form NO_3_^–^ in aerobic soil [8], and by contrast, only a few grow well when taking N only from NH_4_^+^ [4]. This uptake system of N by the roots is under the synchronized control of nutrients availability and hormonal signalling [2,3]. 

Nitrate (NO_3_^−^) plays a signal regulatory role in many physiological processes including root growth. The nutrient uptake efficiency depends on root system architecture in the soil [9], and these processes are controlled by several gene-transcript levels, which are regulated by NO_3_^−^ [10]. NO_3_^–^ regulates root branches under various signalling pathways, such as *NRT1.1* (dual-affinity NO_3_^−^ responsive gene), which is assumed to participate in the nitrate-sensing system [11,12], and also governed by auxin [13]. 

Signalling communication is a general rule rather than an omission. For instance, it is cited that about 1545 genes were the nutrient-related signals of NO_3_^−^, NH_4_^+^, or both nitrogen forms. Also, 982 (64%) genes were controlled by hormonal signals [14]. Several studies reveal that hormone biosynthesis, transport, and transduction are significantly influenced by several mineral nutrients [11]. Hormones play a key role in the root-growth adaptation to NO_3_^−^ readiness [13]. Numerous studies have also shown that NO_3_^−^ regulation of root system architecture (RSA) entails an overlap between NO_3_^−^ and auxin signalling pathways [13,15]. IAA is the most researched and best naturally occurring active auxin [12], and it plays a specific role in the control of systemic inhibition of fresh lateral roots (LRs) developments in response to the sufficient supply of nitrate [16]. Both external NO_3_^−^ and IAA supply significantly influence the auxin concentration in the tiller nodes [17]. As part of the root system, lateral root not only depends on the external NO_3_^−^ supply, but also on the amount of internal NO_3_^−^ supplied to the plant root system [18]. 

Several studies on transporter/channels as well as nutrient responses have been reported for many years. However, at the more basic level, important mechanisms of the signalling overlap between nitrate and auxin in lateral root initiation, emergence, and development in *Arabidopsis* are still poorly understood. In this review, we focus on an in-depth role of NO_3_^−^ in auxin-induced signalling, and the relationship between NO_3_^−^ and IAA transcript levels in the regulation of lateral root initiation, emergence, and development in the model plant *Arabidopsis thaliana*. 

## 2. Classification of Nitrate Responsive Genes 

Root growth and development are influenced by the plants’ nutritious status and the external availability of the nutrients [19,20]. NO_3_^−^ is taken up by the root cell is considered to be a key constituent of the N metabolism. Moreover, the NO_3_^−^ can be subsequently excreted from the root cell by the primary extrusion from the external medium or unloading in the xylem vessel to reach the aerial organs of the plant [21]. For such nitrate transport through the cellular membrane, NO_3_^−^ influx is an active process [21]. The recognizable proof of the proteins and the transport process of NO_3_^−^ inside the plants are essential to understand the components that control NO_3_^−^ retention and distribution within the entire plant. Here, we classify the genes that potentially involved in NO_3_^−^ responses [20,22], as short-term and long-term responsive (Table 1, Table 2 and Table 3), which are described in detail below.

### 2.1. Short Term Primary Nitrate Response (PNR) Genes 

In *Arabidopsis*, *NRT1.1* is one of the chief low-affinity nitrate transporter gene, which affects the root growth in nitrate-responsive signalling, and toggles from the low nitrate condition to high nitrate condition by its phosphorylation of at the Thr101 by CIPK23 kinases (Table 1). *NRT1.1* activates a specific nitrate signalling pathway, thereby regulating RSA, and subsequently stimulating LR growth under low and high NO_3_^−^ supply [55]. It is also evident that auxin might be a secondary signal, triggering the regulatory action of *NRT1.1* on LR growth and development. *NRT1.1* transports nitrate and also encourages the uptake of auxin. In contrast, NO_3_^−^ also inhibits NRT1.1-mediated IAA uptake, which revealed that nitrate signal transduction alters the transport of auxin via *NRT1.1*. Moreover, *NRT1.1* functions in phosphorylation and de-phosphorylation in response to diverse nitrate conditions [56,57] (Table 1). 

In addition to *NRT1.1*, lateral organ boundaries domain (LBD) *LBD37/38/39* have been recently identified to be essential nitrate regulatory genes that suppress several N responsive genes required for NO_3_^–^ uptake and assimilation, and influence NO_3_^−^ content [58,59]. LBDs regulates EXPANSINA 17 (*EXPA17)*, a gene encoding cell wall loosening factor by coupling with *EXPA17* to enhance lateral root emergence. Overexpression of *EXPA17* increases LRs emergence density in the presence of auxin compared to wild type (WT) [60]. Studies have also shown that *LBD18* regulatory gene EXPANSINA 14 (*EXPA14*) expression is involved in the apical tissue of LRs primordium. Collectively, these findings confirm that *LBD18* up-regulates a subset of EXPAs genes to enhance cell division which promotes LR emergence in *Arabidopsis* [61]. Moreover, LBDs act downstream of the auxin influx carrier *AUX1* and *Lax1*, regulating LR initiation and development [62,63]. These transient developmental phases are further synchronized by the action of microRNA (miRNA) families miRNA156 and miRNA172 [20]. It was revealed that miRNA156 targeted SQUAMOSA PROMOTER BINDING PROTEIN-LIKE (SPL) genes *SPL3*, *SPL9*, and *SPL10, which* are involved in the suppression of lateral root growth. This further suggests the function of both miR156 and SPLs in LR development by their response to auxin signalling [22]. *SPL9* was identified to be a sentinel short-term nitrate-responsive gene [57], which is targeted by miR156, while *rSPL9* (overexpression plants of *SPL9*) are resistant to the degradation of miR156 [64]. Thus, the miR156-resistant transgenic plants were identified as the *rSPL9* mRNA, generated from the modified gene resistant to degradation by miR156. This demonstrated the role of the *SPL9* in the regulation of genes involved in the primary NO_3_^−^ response. It was further demonstrated that the role of *SPL9* over-expression on the transcription factor (TF) levels of genes in the network over time leads to a significant increase in *NRT1.1*, *NIA2,* and *NIR1* in response to NO_3_^−^ [57] (Table 1). Moreover, *TAA1* and *TAR2* are involved in auxin biosynthesis, keeps the appropriate root auxin concentration, and the preservation of root stem cell niches (https://www.uniprot.org/). 

From the genome-wide association study (GWAS) analysis, it was revealed that *JR1* (JOSMONATE RESPONSIVE 1) was related to LR length in low and high NO_3_^−^ condition. Compare to high NO_3_^−^ condition, there is a specific defect in the average length of the LRs under low NO_3_^−^ condition. This is an indication that *JR1* functions in regulating LRs length under low N condition [58]. Subsequently, the two genes JASMOANTE RESPONSE (*JR1*) and D AMINO ACID RACEMASE2 (*DAAR2*) are involved in lateral root growth under low nitrate conditions [58,59]. 

Under NO_3_^−^ deficient conditions, *NRT2.4* and *NRT2.5* are down-regulated in the *cbl7* mutants, and therefore accumulate lower NO_3_^–^ content compared to WT plants, and subsequently inhibit the root development upon nitrate starvation in *cbl7* [65], which indicates that *CBL7* enhances root development when expressed in the roots of the young seedlings, which is induced by NO_3_^–^ starvation [65]. In addition, short-term responsive gene family NITRATE-INDUCABLE GRAP-TYPE TRANSCRIPTIONAL REPRESSOR (NIGT1s) transcription factors, incorporated in nitrate starved condition and act as a downstream sensor and transporters of *NPF6.3/NRT1.1* to repress primary root growth in the nitrate sufficient condition [66]. The transcriptase and co-transfection analysis showed that NLPs auto-regulate and control the NIGTs [67]. It is also suggested that *NRT2.1* is independently regulated by NIGTs and *NLP7* because of their different binding sites. Moreover, the *NLP-NIGT1* transcriptional cascade controls the targeted genes together by NLP7-mediated activation, while it independently induces repression [67]. 

The CBL-interacting protein kinases, CIPK8 and CIPK23, are involved in short-term primary nitrate response. Similarly, the subcellular Ca^2+^-dependent protein kinases (CPKs), such as CPK10, CPK30, and CPK32, are necessary for instant nitrate-stimulated cellular and metabolic responses and nitrate-induced root to shoot growth. Under sufficient nitrate conditions, Ca^2+^-sensor CPKs move to the nucleus, whereas *CPK10* targets NLP7S205 phosphorylation to hold *NLP7* inside the nucleus in a nitrate-dependent manner [68,69]. Furthermore, nitrate regulatory gene 2 (*NRG2*) also plays a critical regulatory role in plant primary nitrate responses (PPNR) based on their binding affinity for nitrate [70]. In addition to *NRG2, FPI1* genes (factor interacting with poly (A) polymerase 1) enforce a specific role in nitrate signalling in *Arabidopsis*. *FIP1* is an important part of the polyadenylation factor complex. FIN219-interacting protein 1 (FIP219) have also been demonstrated to be rapidly stimulated by auxin and confined consistently in the cytosol without fluctuation in subcellular localization by light [71]. *FIN219* was exhibited to be a silencer of *COP1* [71], thus demonstrating the role of *FIN219/JAR1* as a significant regulator in modulating auxin-mediated phytohormone signalling. However, its physiological function in light signalling and plant development remains unclear [28]. It was also found that *FIP1* interacts with cleavage and polyadenylation specificity factor 30-L (CPSF30-L), which is also an indispensable player in NO_3_^–^ signalling [29]. *CPSF30* significantly reduced the primary root length and exhibits fewer LRs when *CPSF30* mutant *oxt6* was grown on medium containing IAA [72].

### 2.2. Short and Long Term Nitrate Response Genes 

As stated earlier that *CIPK8* functions as a positive regulator under high and low nitrate conditions [30,40]. The *cipk8* mutant was repressed mainly in NO_3_^–^ limited condition, and its response to both high and low-affinity nitrate signalling system is suggested to be genetically distinct. This indicates that *CIPK8* is involved in low nitrate conditions. Similarly, *CIPK8* also functions in long-term nitrate modulated primary root growth and nitrate-induced expression of a vascular malate transporter [30,73]. 

Furthermore, the transcription factors belonging to the bZIP family, namely *TGA1* and *TGA4,* are stimulated by NO_3_^–^ in the *Arabidopsis* root [31]. *TGA1* and *TGA4* regulate the expression of NO_3_^–^ transporter genes *NRT2.1* and *NRT2.2* [31]. The stimulation of both *TGA1* and *TGA4* is repressed in *chl1-5* and *chl1-9* mutants after NO_3_^–^ application, which indicated that both *TGA1* and *TGA4* are regulated by nitrate transport, rather than the signalling function of *NRT1.1* [31]. These findings suggest that *TGA1* and *TGA4* act as a primary nitrate responsive genes (Table 2).

Another short-term and long-term nitrate responsive transcription factor, *NLP7* plays a prominent role in the LRs growth in response to nitrate, promotes the LRs density in high NO_3_^−^ condition, acts as nitrate sensing and assimilation, suggested to be short-term PNR gene. However, the *nlp7* mutant exhibited lateral roots inhibited phenotypes on split-root plates in both high and low nitrate conditions, further suggesting that *NLP7* may mediate local nitrate response [23,42]. Conversely, the present findings indirectly strengthen the possible function of the *NLP7* in regulating the LRs growth in varying nitrate conditions, which suggests the role of *NLP7* as long-term nitrate response gene [50]. In addition to *NLP7*, the transcription factor that regulates the expression of the prototypical NO_3_^–^ response gene is *SPL9* [57].

### 2.3. Long Term Nitrate Response Genes

When the supply of nitrogen becomes limited, the long-term high-affinity type transporters enabling the nitrate uptake, are expressed in the root to enhance the efficacy of nitrogen acquisition [45,46] (Table 3). Among them, the nitrate inducible MADS family protein ANR1 promotes lateral root in response to nitrate [60]. *NRT1.1* and its downstream MADS-box transcription factor *ANR1* mediate local signalling [32,74]. In the long-term mechanism, *ANR1* functions as a positive regulator in the signalling pathway of nitrate-induced LRs proliferation [75,76].

Another long-term high-affinity nitrate regulatory gene *NRT2.1* involved in the NO_3_^−^ uptake, are responsible for the accumulation and induction of the transcript in the root under low nitrate conditions [77]. Similarly, microRNA (miRNA) emerged as a key regulator in nitrate-induced root growth and development. miR167 targets and regulates the expression of auxin response factor (*ARF8*), and both *ARF8* and miR167 are expressed in the root cap [43,78]. *ARF8* is accumulated in the pericycle by inhibition of miR167 under nitrate availability and subsequently display an increased ratio of initiation vs. emerging LRs in response to NO_3_^−^ treatments [42]. Moreover, miR393 was activated in response to nitrate supply, mainly by binding the auxin *AFB3* transcript and subsequently modulating the accumulation of AFB3 mRNA in the root in response to nitrate treatments [79].

In *A. thaliana* plants, *CLE41*, a homolog of the phloem-derived secretory peptide tracheary element differentiation inhibitory factor (TDIF) [45], and the CLE41 peptides (CLE41p) stimulated the proliferation of vascular cells, despite the delay in its division into phloem and xylem cell lineages. *CLE19/41/44/TDIF* are localized in the vascular stem cells and function in the maintenance of LRs emergence [80,81]. In response to high nitrate availability, *HNI9/AtIWS1* (HIGH NITROGEN INSENSITIVE9) plays an important role in the systemic regulation of root NO_3_^−^ uptake in *Arabidopsis* [32], and functions as an inhibitor of *NRT2.1* transcription in the root [82]. Moreover, it plays a significant role in N signalling by regulating hundreds of NO_3_^−^ responsive genes in the root [82]. Among other, long-term nitrate responsive TFs, that function in lateral root development of *A. thaliana*, CLEs-CLVs related peptides function as a regulatory module in the nitrate signalling pathway and negatively regulates LRs growth under limited nitrate supply [80,83]. Similarly, CEP hormones, 15-amino-acid peptides, act as negative regulatory hormones in growth and development. *CEP1* is a candidate for a novel peptide plant hormone, mainly expressed in the lateral root primordial, and its overexpression significantly inhibits root growth [80]. These polypeptides are imported into the roots to activate nitrate transporter genes in the rhizosphere under a sufficient supply of NO_3_^−^ [81]. In addition, *AtTCP20* is also another nitrate responsive transcription factor, which modulates LRs in nitrate dependent manner, and is studied in chrysanthemum and heterologous overexpression in *Arabidopsis*. The overexpression of *CmTCP20* significantly increased the number and average length of lateral root (LRs) compared with the wild type in chrysanthemum and *Arabidopsis* [78], indicating that *TCP20* is regulated by NO_3_^−^, supplied to N starved roots.

Members of the bZIP transcription factor family, *TGA1* and *TGA2*, are induced by nitrate in the roots [31]. Transcriptome analysis of roots showed that differentially expressed numbers of genes regulated by nitrate availability suggest *tga1tga4* as a double mutant. Moreover, *TGA1* and *TGA4* significantly decrease the induction of its target genes *NRT2.1* and *NRT2.2*. Further findings showed that *TGA1* could bind *NRT2.1* and *NRT2.2* and regulates their expression [42,84]. As stated earlier, adequate NO_3_^−^ suppresses the level of miR167a to allow the accumulation of *ARF8* transcript in the pericycle upon nitrogen treatments, while under low NO_3_^−^ condition miR167a targets *ARF8* [23]. Similarly, the *AFB3* transcriptional factor plays a significant role in coordinating LRs and primary roots under adequate nitrate supply. Nitrate also activates *NAC4,* as downstream of *AFB3*, which is an essential part of regulating the nitrate-responsive system, the mutants of *NAC4, nac4* showed altered LR growth. Meanwhile, the primary root growth remains normal under nitrate state. Thus, *AFB3* regulatory network confers changes in LRs development in response to nitrates [49], which confirms the long-term role of these transcripts in response to NO_3_^−^.

Similarly, the complex overlap between nitrate and phytohormone auxin signalling pathways are regulated by genomic distinctiveness. This induces the allelic variety of genes. ROOT SYSTEM ARCHITECTURE 1 (RSA1) and PHOSPAHT TRANSPORTER 1 (*PHO1)* are the two genes regulating allometric parts of root system architecture (RSA), however the mechanisms through which their NO_3_^−^ and IAA signalling pathways regulate the root architecture remains to be understood [82].

## 3. Nitrate Responsive Genes Enhances Auxin Activity in the Roots

Nitrate (NO_3_^−^) supply can alter auxin biosynthesis and its transport [40,85,86]. Indole-3-acetic acid (IAA) is an essential plant native auxin [53], which is required for plant growth and development under diverse environmental conditions [87]. In *Arabidopsis* roots, the decay of auxin cannot be achieved by the translocation of auxin from shoot to root, which indicated that shoot localized auxin alone is not adequate for supporting the root initiation, elongation, and development [84]. Auxin transportation has been hypothesized to be crucial intercellular and intracellular IAA distributions in the plant. Inside the plant cells, it was reported that for the directional anionic auxin and formation of the polar flow, it needs transporters and their uneven localization [88]. This evidence revealed that roots nitrate transcriptionally regulate the transport and biosynthesis of auxin [89].

To date, few transcription factors that contribute to NO_3_^−^ dependent auxin efflux have been studied. For instance, nitrate responsive genes which include *NRT1.1, NRT2.1, NRT2.2, NIA1, NIA2*, *NIR,* and Arabidopsis Nitrate Regulated (*ANR1*) [75]; *TAR2* (tryptophan aminotransferase related 2) [90]; NIN like Protein 6 (NLP6) [91]; NIN-Like Protein 7 (NLP7) [92]; LOB Domain-Containing proteins (LBD37/38/39) [62]; Squamosa Promoter Binding Protein-Like 9 (SPL9) [93]; Basic Leucine-Zipper 1 (*bZIP1*) [94]; NAC Domain Containing Protein 4 (NAC4) [49]; *TGA1/TGA4* [31], Teosinte Branched1/Cycloidea/Proliferating Cell Factor 20 (*TCP20*) [78]; and Nitrate Regulatory Gene 2 (*NRG2*) [70]. Moreover, the link between *NLP6* & *7*, *TGA1*, *bZIP1*, and *TCP20* with the promoter of the genes of interest was also confirmed [87].

**Figure 1:**
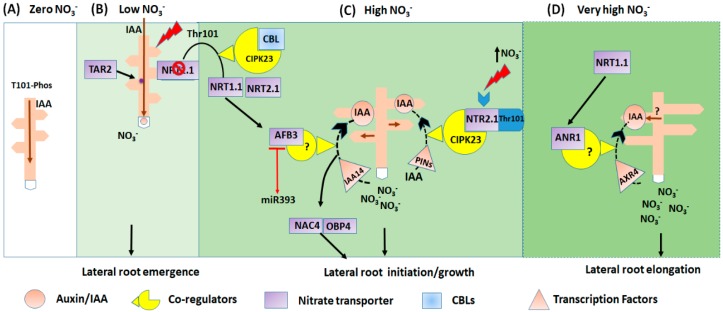
NO_3_^−^ regulates auxin activity, and consequently controls the NO_3_^−^ assimilation pathway and transport. The model represents the root morphology subjected to four different nitrate conditions. (**A**) The deficient (zero) NO_3_^−^ stimulates auxin translocation from shoot to roots. (**B**) This mechanism is utilized under low NO_3_^−^ conditions, either *NRT1.1* transport auxin from shoot to root, causing LRs emergence, or reduced the auxin storage in primordia and new lateral root tip, and subsequently inhibits LRs emergence and elongation. Thus considered as the principal regulator for the auxin biosynthesis in the root promotes lateral root growth. (**C**) Under the high nitrate NO_3_^−^ condition; *NRT1.1* induces the expression of *AFB3*, and the AFB3-dependent auxin signalling, regulates root growth. The *AFB3-NAC4-OBP4* signalling network expressed their protein in the root pericycle of the cell. The *NAC4* and *OBP4* part of the pathway is probably regulated by AUX/IAA protein IAA14. The black dotted arrow indicates the effect of *AFB3* on the IAA accumulation, promotes lateral root initiation under high nitrate condition. (**D**) Under the excessive supply of NO_3_^−^; *NRT1.1* stimulates the ANR1-dependent signalling pathways that modulate lateral root elongation. The black dotted arrow indicates the effect of *NRT1.1* on the IAA accumulation and expression of *ANR1* in the LRs primordial, and lateral root development. The question mark represents the unconfirmed effect of *NRT1.1* nitrate transport activity on the expression of *ANR1* [97].

It has been reported that in the roots *TAR2* expression is significantly increased compared to *TAA1,* which is induced moderately under low NO_3_^−^ condition. However, in shoots, both genes were repressed. *TAR2* was expressed in the root pericycle and also the vasculature of the root maturation zone close to the root tip [85] (Figure 1). The mild NO_3_^−^ deficiency positively affects LRs formation in *Arabidopsis* which required an auxin biosynthesis gene *TAR2*. A change L-Trp to indole-3-pyruvic acid (IpyA) by *TAR2* is the first step in the IpyA pathway branching from a Trp-dependent auxin pathway [90]. Under low NO_3_^−^ source, the *TAR2* expression was up-regulated, resulting in an increase in IAA levels in the developing LRs. Nevertheless, the *tar2-c* null mutants exhibit much shorter total LR length and fewer visible LR numbers, demonstrating that mutation of *TAR2* reduced the LR formation [85]. Therefore, low NO_3_^−^ stimulated LR emergence depended on root-synthesized auxin in a TAR2-dependent manner [50]. So far, all the NRTs-dependent hormone transporters are involved in the local redistribution of the hormones within the plant [40]. NO_3_^−^ transporter enhances the redistribution and transport of auxin at a short distance [95,96]. For instance, *NRT1.1* transports auxin (IAA), and consequently overlaps nitrate and auxin (IAA) signalling pathways [40]. *NRT1.1* transports the auxin from the lateral root primordial under low nitrate condition, thus suppressing the development of LR primordia and fresh LRs. However, at optimum nitrate condition, the repressed NRT1.1-dependent IAA transport was activated, which result in subsequent accumulation of auxin in the LRs and promotion of the primordium growth [52,81]. Under the low N condition, the *nrt2* mutant suppresses the lateral root emergence and the IAA storage in the LRs primordia [85].

*NLP7* is the primary auxin-efflux protein’s carrier localized in the plasma membrane [44]. NLP7-induced auxin efflux activates *PIN7* [88]. Further studies have shown that *NLP7* encodes 851 genes under optimum nitrate conditions. In addition, new nitrate regulatory factor NITRATE REGULATORY GENE 2 (*NRG2*), disrupts the stimulation of NO_3_^−^ sensor genes in the *nrg2* mutant when subjected to nitrate treatments. This indicates the physical interaction of *NRG2* with *NLP7* in the nucleus [87]. Similarly, NIGT1s expression is auto-regulated and controlled by NLPs. Therefore, NIGT1s inhibit the *NRT2.1* stimulation by *NLP7* [67]. *NLP7* regulates the fluctuation in local auxin by controlling the auxin-efflux to build up and sustain the root primordium and determine the LRs number [88]. Genome-wide transcriptional profiling data revealed that *NLP7* and *TAR2* are among the top NLP7-activated genes, in addition to the nitrate inducible genes such as *NiR*, *NIA1*, *FNR2*, and *NRT2.1* [32,78]. A transcriptomic study demonstrates that an auxin bio-module containing auxin carrier 9 (including among others *PIN1*, *PIN2*, *PIN4*, and *PIN7*) is specifically regulated by NO_3_^−^ [98]. Furthermore, it suggests TCP20-NLP6/7 complexes as the upstream target of the Auxin- ROP2-TOR-E2Fa/b signalling pathway [89].

In *Arabidopsis* root, a nitrate responsive miR393/AFB3 regulatory module, which assimilates nitrate and auxin signals control both primary and LRs growth [49], and is capable of stimulating the nitrate (5 mM) signal to promote root growth in response to auxin. Independent of nitrate metabolism and transport, AFB3 induces auxin signalling, shows sensitivity and response to NO_3_^−^, subsequently regulate LRs growth and functions as downstream of nitrate signalling [49]. Family of no apical meristem (NAM)/activating transcription factors/cup-shaped cotyledon (ATF/CUC) proteins, and it’s targeted genes *OBP4*, acts as a downstream target of *ABF3* gene. The interactive signalling network of *AFB3-NAC4-OBP4* with their existing protein expression in the pericycle cell of the root is required for the nitrate inducible LRs initiation and emergence (Figure 1). The part of *NAC4-OBP4* signalling pathways is possibly regulated by AUX/IAA proteins, IAA14 [49,99] (Figure 1).

Under nitrate deficient condition, the AGL17-clade MADs-box gene, *AGL21,* and auxin promote longer LRs in *Arabidopsis*. However, the overexpression (OE) of *AGL21* significantly up-regulates the auxin biosynthesis genes *YUC5*, *YUC8*, and *TAR3,* and is down-regulated in *agl21* mutants. *Ag121* mutants showed a decrease in the rate of LRs elongation in low nitrate supply, demonstrating that *AGL21* promotes the local auxin biosynthesis in the LR primordia and LRs, thus positively regulating LRs development [100].

Studies have shown that *SPL* may function in the auxin homeostasis. To this end, OE of *SPL* led to the downregulation of auxin reporter DR5-GUS, and subsequently down-regulate several auxin-responsive genes in the leaves of *spl-D*. Further studies have shown that the two auxin biosynthesis genes, *YUC2* and *YUC6* were significantly suppressed in *spl-D* plants. Both genomic and phenotypic studies on *spl-D/yuc6-D* double mutant revealed that *SPL* might control auxin homeostasis by suppressing the transcription of *YUC2* and *YUC6* and contributing to lateral organ morphogenesis [48].

The report of Cui-Hui Sun showed that under low NO_3_^−^ condition, the nitrate signalling pathway has a specific stimulatory effect on the LRs growth. The molecular players’ complexes involved in these pathways regulate various stages of LR development and also influence auxin biosynthesis and transport [50]. These findings demonstrate that nitrate (NO_3_^−^) signalling constituents function upstream of auxin biosynthesis, transport, and control of LR development.

## 4. Nitrate Signalling Pathways and Auxin Response

The NO_3_^−^ and IAA cross-talk was first identified by Goerge S. Avery and Louise Pottorf in the 1940s. They demonstrated a direct relationship between NO_3_^−^ and IAA supply [101]. Numerous studies have also revealed that the interaction between NO_3_^−^ and the auxin signalling pathways that regulate RSA [39]. Presently, the molecular mechanism of NO_3_^−^ signalling transduction has been revealed in *Arabidopsis* [43]. NRT1, NRT2, CLC (Chloride Channel), and SLAC1/SLAH (slow anion channel 1/ SLAC1 homolog) are the four nitrate transporters families that have been characterized in *Arabidopsis* [102].

### 4.1. Nitrate Uptake

**Figure 2:**
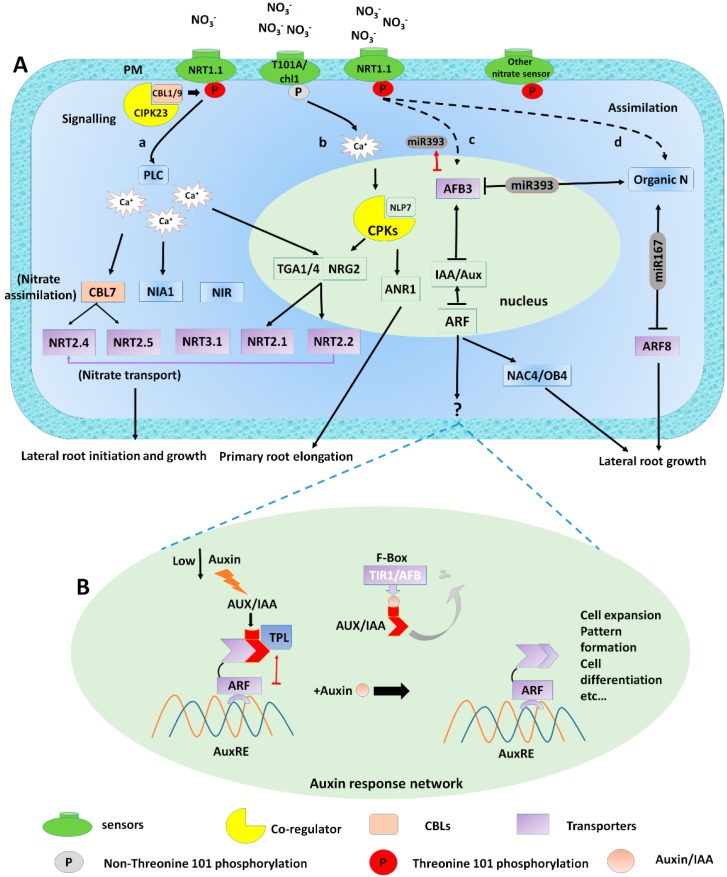
Signalling mechanism of nitrate mediated auxin modulation induces lateral root growth; (**A**) (**a**) *NRT1.1* initially senses, taken up and transport by the *NRT1.1* transporter, thereby changing its uptake affinity and modulating phosphorylation to activate signalling pathway. In low nitrate conditions, the phosphorylation switches *NRT1.1* into the high-affinity system. This sensing ability of NO_3_^−^ altering *NRT1.1* phosphorylation causes calcium (Ca^2+^) efflux by the activation of phospholipase C (PLC). This results in varying the expression of (TGA1/4*) and nitrate transporter genes (*NRT2.1, NRT2.2, NRT3.1*) and nitrate assimilation genes (*NIA1* and *NiR*). (**b**) *T101A/chl1* mutant triggers Ca^2+^-ANR1 signalling from the endosomes under high NO_3_^−^ supply. (**c**) Under adequate NO_3_^−^ supply, *AFB3* regulates *NAC4* and *OBP4* expression, which as a result affect the root renovation. (**d**) Finally, as a result of nitrate assimilation organic nitrogen is produced, this system includes miR167 and miR393, which regulates *AFB3* and *ARF8*, respectively (**B**) In Auxin response network; Class ARF directly connects to the AuxRE (red arrow). Under low nitrate conditions, AUX/IAAs and TPLs repress transcriptional activation by class-ARFs. The degradation of Aux/IAA released this inhibition by connecting auxin and TIR1/AFB. (See text for further details).*TGA1/4 regulatory factors mediate nitrate response inside *Arabidopsis* root, and while the link of *TGA4* to PLC is not approved. Other transcription factors, for instance, *NRG2*, *NLP7*, connection to Ca^2+^ signalling is currently unknown.

*NRT1.1* is phosphorylated by the CBL1/9-CIPK23 complex [102,103]. Nitrate is absorbed into the root cell by plasma membrane-localized transporter family, NRT1 and NRT2 [104]. Under limited nitrate condition, *CBL1/9* interacts with *CIPK23* to form the CBL1/9-CIPK23 complex, which phosphorylates *NRT1.1* at the threonine site T101, for efficient NO_3_^−^ transport [104,105] (Figure 2). This phosphorylation promotes *NRT1.1* enrolment into functional membrane microdomains at the plasma membrane (PM). This activity enhances the NRT1.1-dependent auxin flux, and as a result, drains the auxin level from the LR tips and limits growth. When the NO_3_^−^ supply was increased, the non-phosphorylated *NRT1.1* shows oligomerization, reduced lateral mobility at the PM and exhibits inducible endocytosis. This activity promotes LR growth, induces NRT1.1-auxin transport activity at the PM, and stimulates Ca^2+^-ANR1 signalling from the endosomes [54].

The phosphorylation activates the high-affinity nitrate transporter *NRT2.1* [106], and stimulation of *NRT1.1*, *NRT2.1*, *NRT2.2*, and *NRT2.4* was reported under nitrate starved seedlings after nitrate supply and all the nitrates assimilation genes were up-regulated [107,108]. Studies have identified the roles of the Ca^2+^ in the nitrate signal transduction and stimulate nitrate-inducible regulation of the genes expressed in *Arabidopsis*. It was observed that nitrate treatments significantly increased cytoplasmic Ca^2+^ levels in the roots and the seedlings of the plants [109] (Figure 2). *NRT1.1* is governed by two modes of phosphorylation at Th-101 residue to regulate LRs growth, modulating NO_3_^−^ dependent basipetal auxin transport and NO_3_^−^ induced signal transduction. By using the two *Arabidopsis* phosphomimetic *NRT1.1T101A* and non-phosphorylatable *NRT1.1T1101D* mutants respectively [54], *NRT1.1T101D* has presented the fast lateral mobility and membrane partitioning that enabled auxin flux under low-nitrate conditions while In contrast *NRT1.1T101A* mutants showed low lateral mobility and oligomerized at the plasma membrane (PM), where it induced endocytosis under high NO_3_^−^. These activities stimulate LR development by suppressing NRT1.1-mediated auxin transport on the PM and inducing Ca^2+^-ANR1 signalling network from the endosomes [54] Figure 2A. To this end, phosphorylation mediates the calcium flux in the PM by NO_3_^−^ transporter *NRT1.1*, subsequently regulates auxin flux and nitrate signalling in lateral roots development [54]. These studies indicated that NO_3_^−^ mediated Ca^2+^ flux modulating auxin response and polar auxin transport in the root growth and development.

### 4.2. Auxin Response Network

It was previously reported that auxin-NO_3_^−^ pathway by identifying a passive feed-forward mechanism consisting of auxin receptor auxin signalling F-box 3 (AFB3) and microRNA miR393 [79]. The expression of AFB3 protein in response to NO_3_^−^, indicating Ca^2+^-independent pathways that regulate the nitrate-sensitive genes [109] (Figure 2A). Under high nitrate conditions, *AFB3* is stimulated, whereas, under lower NO_3_^−^, miR939 induced by nitrate reduction and assimilation which produces N metabolites, and subsequently *AFB3* suppressed by miR393 [79] (Figure 2A). Furthermore, it was established that nitrate-AFB3-NAC4-OBP4 mediated perception, signalling and response of auxin in a nitrate-dependent manner is mainly regulated by multiple signalling mechanisms and their coordination under unfavorable nitrate condition [68]. Nitrate-specific induction of *AFB3* inside the root may regulate a specific signalling network of Aux/IAA and *ARF* factors that modulate *NAC4* activation. AFB3 regulates the transcription of the IAA-responsive gene by promoting the degradation of the Aux/IAA transcriptional repressor of auxin [110] (Figure 2A). The microRNA, miR167, and its target auxin-responsive factor *ARF8* mRNA [42] function to regulate several genes connected via a network and activate the lateral root initiation and inhibition of elongated roots in response to NO_3_^−^ [42] (Figure 2A).

Auxin stabilizes the communication among TRANSPORT INHIBITOR RESPONSE1/AUXIN SIGNALLING F-BOX proteins (TIR1/AFB3) and Domain II of AUXIN/INDLOE-3-ACETIC ACID (AUX/IAA) transcriptional co-regulators to stimulates the ubiquitin-dependent breakdown of AUX/IAA protein in the 26S proteasome [111] (Figure 2B). When the auxin concentration is low, individuals from the AUXIN/IAA-INDUCIBLE (AUX/IAA) family of transcriptional repressors bind with DNA-binding protein of ARF [112,113], which exactly possess auxin-response promoter elements (AuxREs) in various auxin-regulated genes [114,115]. AUX/IAA protein inhibits the *ARF* function either by passively sequestering ARF protein away from their specific target promoters [116], or by interacting AUX/IAA with the co-repressor TOPLESS (TPL) to stimulate chromatin inactivation and silencing of *ARF* target genes [117]. The auxin concentration is increased by the auxin-stimulated module of co-receptor complexes, which include TIR1/AFBs family and an AUX/IAA member [118]. TIR1/ABFs specifically allows subunit of nuclear S-PHASE KINASE ASSOCIATED PROTEIN 1-CULLIN-F-BOX PROTEIN (SCF)-type E3 ubiquitin-protein ligases (SCFTIR/AFB) and stimulate the recognition of substrate. The auxin response is initiated by connecting the hormones to the TIR1/AFB receptor. The auxin receptor is a constituent of the SCFTIR1/AFB ubiquitin ligase complex [119]. This complex binds auxin to its receptor TIR1/AFB to activate the formation and breakdown of the polyubiquitination of AUX/IAA repressor, and subsequently activates ARF that induces auxin-responsive gene transcription [120,121,122] and represents the pivotal role of auxin signalling (Figure 2).

In a nutshell, auxin-initiated AUX/IAA removal regulates *ARF* repression and activates the transcription of primary genes. [123]. The abundance of Aux/IAA-ARF modules chronologically generate new LRs and control LRs development in *Arabidopsis* model plant.

## 5. Relationship between Nitrate Level and Auxin Translocation between the Root and the Shoot

**Figure 3:**
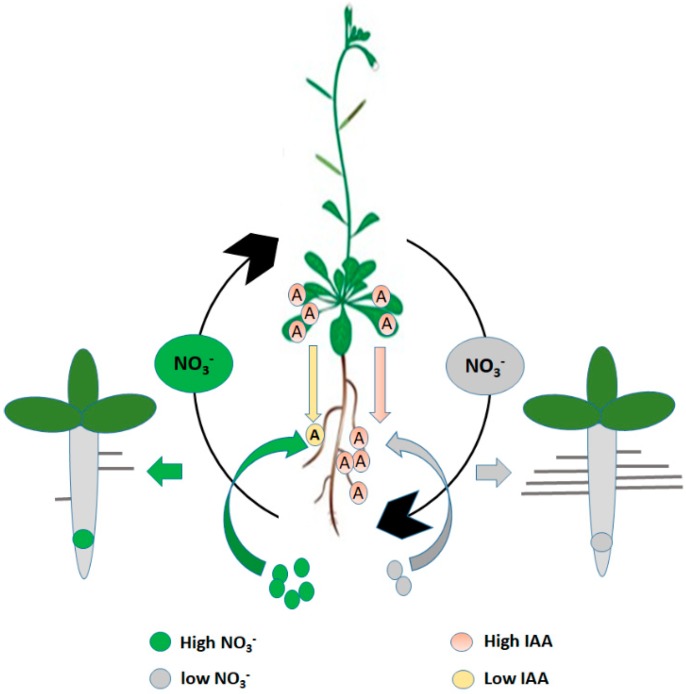
The schematic model represents NO_3_^−^ dependent shoot-root auxin transport and accumulation inside the *Arabidopsis* root. “A” represents auxin (IAA).

A decrease in NO_3_^−^ supply tends to promote auxin translocation from shoots to roots [124] (Figure 3). Here, we discuss the mechanism involved in the transportation of the auxin in response to different NO_3_^−^ concentrations and the influences of the auxin concentration on the root growth.

### 5.1. Auxin Translocation in Response to Nitrate Level

High NO_3_^−^ concentration (50 mM) negates the lateral root growth [121]. These responses are linked with an auxin transport inhibitor. Previous studies have established that the internal supply of nitrate reduced shoot to root auxin transport and subsequently decrease root auxin concentration to a significant level for lateral root growth. Therefore, for the stimulation of the lateral root growth, change in the root auxin concentration alone is not adequate, as nitrate concentration is also a determinant factor [121,125]. There is a decrease in the level of auxin in the root when there is an adequate supply of nitrogen because the adequate supply of NO_3_^−^ seems to inhibit auxin transport activity from the shoots to the roots. Furthers reports have shown that the external IAA concentration partially lowers the stimulatory effect of localized nitrate (Figure 3). This inverse relationship between nitrate and auxin concentration disrupts the LRs development [126].

As stated earlier, the auxin level in the roots is reduced by an adequate supply of nitrate (NO_3_^−^) which disrupted LRs development [126]. Moreover, The tissue auxin fixation was estimated in the roots 24 h after transferring the *Arabidopsis* seedlings from higher to lower nitrate medium [16]. Results indicated that the restricted root growth induced by 50 mM NO_3_^−^ is replenished back to its normal growing state, 24-48 h after reducing the high nitrate (NO_3_^−^) concentration. It is worthy to note that if the auxin plays a role in controlling this process, its concentration in the root may depend on the time before activating the inhibited lateral root. Further revealed that, a 50% increase was observed in the IAA content of the root when moved to 1 mM NO_3_^−^ compared to that of 50 mM NO_3_^−^ [127]. Similarly, the seedlings grown under 1 mM NO_3_^−^ has a root auxin concentration that was four-fold more than the plants grown on 8 mM NO_3_^−^ condition [128]. This is also evident that high NO_3_^−^ (8 mM) concentration significantly reduces the root auxin content [128]. Meanwhile, the high nitrate supply reduces the IAA concentration in the phloem exudates. Thus, the suppression of root growth by high nitrate could be linked to the reduced IAA level in the roots, particularly in the root tip region. It is speculated that the inhibitory effects of high nitrate concentration on the restricted root growth might be related to the decline in auxin content of the root [126].

### 5.2. Influence of Auxin Concentration on Root Growth

Recent studies demonstrated that auxin accumulation in the LRs primordia overlaps with the low nitrate-stimulated expression of *TAR2* in the root [16,85]. The application of the 2-4-carboxyphenyl-4, 4, 5, 5-tetramethylimidazoline-1-oxyl-3-oxide (cPTIO) under NO_3_^−^ supply, initiates auxin (IAA) level in the root [112]. In contrast to wild-type (WT), the *ospin1b* mutant has lower auxin level in their root, fewer LRs and shorter seminal root (SRs). These results demonstrate the effect of NO_3_^−^ on LR arrangement and seminal root (SR) elongation by regulating auxin transport and NO_3_^−^ [112]. NO_3_^−^ and IAA regulate the expression of the rice adenosine phosphate isopentenyltransferase (*OsIPT)* genes, limit the cytokinin (CTKs) biosynthesis in the tiller nodes, thereby regulating the development of tiller bud in rice [17]. Similarly, it is revealed that there was no increase in LRs elongation of auxin resistant *axr4* when subjected to low nitrate conditions [113]. Reversed-phase ultra-performance liquid chromatography (RP-UPLC) results on the plants showed that LR auxin concentration was increased while the nitrogen level decreased, hence the tea plant LRs formation might be stimulated by low nitrogen level via IAA biosynthesis and accumulation [129].

Auxin exhibits either a stimulatory or inhibitory effect on the primary and lateral roots of the plants depending on auxin concentration in the plants [130]. A reduced IAA (auxin) level in the roots inhibits LRs development [90], which is an indication of the NO_3_^−^-regulated auxin transport in the root. It has been inferred that NO_3_^−^ increase in the shoot may repress the transition of auxin to the root, resulting in auxin-transport for LRs development [130]. However, in the auxin signalling pathway, high nitrate enhances LR development by influencing auxin levels in the roots. For example, at high external nitrate fixation, the LRs elongation in maize was repressed, due to a decrease of auxin translocation in phloem from shoot to root [90].

## 6. Nitrate Responsive Regulatory Module Initiates Root Growth

**Figure 4:**
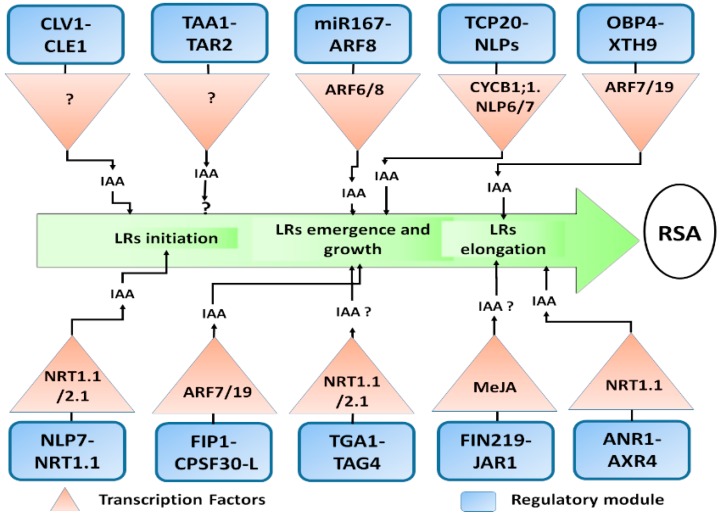
Molecular players involved in nitrate signalling regulating the primary and lateral roots; Schematic presentation of a regulatory module involved in the nitrate-mediated auxin response in *Arabidopsis* root. For clarity purposes, all players and their secondary transcription factors (both upstream and downstream) are presented with blue and black colors respectively, which might not be the case in a plant cell. Auxin fluxes and accumulations contribute to primary and lateral root growth are indicated with the black arrow. However, the detailed mechanism and other transcripts are still unknown.

Nitrate modulates universal gene expression [131] (Figure 4), and some of the nitrate responsive regulatory modules are discussed here.

### 6.1. TAA1 and TAR2 Regulatory Module

Analysis of *TAA1* and paralogues revealed a link between assimilation of local auxin, tissue-specific ethylene effect, and organ development. Indole-3-pyruvate (IPA) processed auxin synthesis is key to synthesize auxin response to light and environmental cues [36]. *TAA1* and *TAR2* are interlinked with auxin synthesis, required for keeping the suitable root auxin fixation and also required for the protection of root stem cell niches (https://www.uniprot.org/).

### 6.2. ANR1 and AXR4 Regulatory Module

There is an interaction between the *ANR1*-dependent pathway and auxin signalling [113]. However, it remains unclear whether there is a relationship between the *ANR1* and the miR393/AFB3 module. It has been reported that *NRT1.1* functions upstream of *ANR1*, as it regulates LRs elongation, obviously by its role as NO_3_^−^ sensor [55]. In addition, NO_3_^−^ signal activated by NO_3_^−^ sensor (targeted at the PM), is transmitted by pathways that consist of the products of *ANR1* and *AXR4* genes. The possible function of *AXR4* and whether it lies upstream or downstream of *ANR1* in a signal transduction pathway remains unclear [96].

### 6.3. TGA1 and TAG4 Regulatory Module

Using the integrative bioinformatics approach, *TGA1* and *TGA4* TFs were shown to mediate NO_3_^−^ responses in *Arabidopsis* roots. The ATH1 Affymetrix microarray analysis showed that *tga1/tga4* double mutants have different responses to nitrate compared to wild type. This implies that TFs regulate genes that participate in the NO_3_^−^ transport and metabolic processes. Also, ChIP analysis indicates that the TFs control the expression of high nitrate affinity transporters *NRT2.1* and *NRT2.2* by binding to their promoters. This reveals that *TGA1/TG4* & *NRT2.1/NRT2.2* regulates the lateral root growth induced by nitrate signalling in *Arabidopsis* [31].

### 6.4. CLV1 and CLE1 Regulatory Module

This signalling module consisting of the nitrogen responsive (CLAVATA3/ESR-related) and leucine-rich occurring receptor-like kinase CLAVATAI (CLV1) are expressed in the root vasculature of *Arabidopsis* root [132]. *CLV1* and *CLE1* regulate the elongation of the root system under limited nitrogen conditions [132]. *CLE1, -3, -4* and *-7* stimulated by limited internal NO_3_^−^ in the root, and mainly expressed in the cells of root pericycle [133]. In *A. thaliana* plants, the *CLE41* peptides (*CLE41p*) promote the proliferation of vascular cells. This proliferation is dependent on auxin binding since it was improved by the exogenous application of synthetic auxin. This report established that vascular architecture is regulated by various *CLE* peptides related to hormonal signalling [74].

### 6.5. FIN219-JAR1 Regulatory Module

*FIN219/JAR1* plays an exemplary role in regulating the combination of phytohormones in an auxin-dependent manner [28]. Specifically, Since *FIN219* contains far-red (FR) light and methyl jasmonate (MeJA), that control various TFs including 94 basic helix-loop-helix (*bHLH*) TFs. Some of the loss of function mutant *bHLH* influenced by *FIN219* demonstrate an altered response to MeJA in the regulation of the hypocotyl and root elongation. Thus, *FIN219/JAR1* is specifically controlled by exogenous MeJA and interacts with different plant hormones to modulate the hypocotyl and root elongation of *Arabidopsis* seedlings, likely by regulating a group of TFs [118].

### 6.6. FIP1 and CPSF30-L Regulatory Module

It is reported that *FIP1* interacts with the cleavage and polyadenylation specificity factor 30-L (CPSF30-L), which is also a fundamental player in nitrate signalling [29]. To study the expression of *CPSF30* in the LRs by qRT-PCR plant poly (A) signal (PAS) in *oxt6* was performed. The results demonstrated that in the absence of auxin the expression of *ARF7* and *ARF19* was found lower in *fip1* compared to WT, which may be one of the reasons for less LRs in *oxt6*. However, in *oxt6,* there was a significantly greater increase in the expression of *ARF7* and *ARF19* when exogenous IAA was applied than that of WT. This further validates the role of *CPSF30* in auxin regulation of LRs growth and development to a greater extent through *ARF19* [134].

### 6.7. TCP20-NLPs Regulatory Module

The branched1/cycloidea/proliferating cell factor1-20 (*TCP20*) and NIN-like protein (NLP) NLP6 & NLP7, which functions to induce nitrate assimilatory genes, target the promoter upstream of nitrate reductase genes *NIA1* [79,135]. *TCP20-NLP6* and *7* heterodimers aggregate in the nucleus, and its overlaps mediate up-regulation of NO_3_^−^ assimilation genes, and down-regulates G_2_/M cell-cycle marker gene *CYCB1;1* [79]. In a likewise manner, TCP20-NLP6 & 7 supports root meristem development under N deficiency. This report elucidates mechanisms through which plants facilitate accessibility to nitrate responses, subsequently interconnecting NO_3_^−^-assimilation and signalling with cell-cycle progression [79]. It was revealed that Chrysanthemum morifolium *TCP20* (*CmTCP20*) positively influence auxin accumulation in the LRs, partially by improving auxin biosynthesis, transport, and response, and thereby stimulating LRs growth [78].

### 6.8. Nitrate-Responsive OBP4-XTH9 Regulatory Module 

Xyloglucan endotransglucosylases (XTHs) plays a crucial role in cell wall biosynthesis of group genes 1 *XTHs* gene, e.g., *XTH1-11*. *XTH9* is highly expressed in the LRs primordial. Consistently, *xth9* mutant shows fewer LRs compared to the OE *XTH9* exhibits more LRs. This indicating the potential role of *XTH9* in regulating LRs growth [122]. Genetic analysis revealed that this function of *XTH9* depend on auxin-mediated *ARF7/19* and the downstream *AFB3* in response to nitrogen signals, stimulates the expression of *XTH9* to enhance the LRs [122].

### 6.9. Nitrate Responsive NLP7 and NRT1.1 Module

It was reported by [136], that the expression of a transceptor *NRT1.1* is modulated by NIN-like protein 7 (NLP7). Genetic and molecular analyses indicated that *NLP7* functions as an upstream of *NRT1.1* in the regulation of nitrate when NH_4_^+^ is present, while in absence of NH_4_^+^, it functions in nitrate signalling without being mediated by *NRT1.1.* However, in the *nlp7* mutant, the expression of *NRT1.1* initiates a partial or complete nitrate signalling [136]. More convincing evidence from ChiP and EMSA analyses indicated that *NLP7* may bind to the particular region of the promoter of *NRT1.1*. Hence, *NLP7* gene plays a significant role in NO_3_^−^ signalling by regulating *NRT1* [136].

## 7. Conclusions and Future Perspective

Recently, the system biology approach was established to distinguish the constituents and the crosstalk between nitrate and hormonal signal influenced by nitrate-signalling [137]. NO_3_^−^ and IAA contribute to the cascade of events involved in the signal transduction towards the auxin-induced adventitious and LRs formation [138,139].

**Figure 5:**
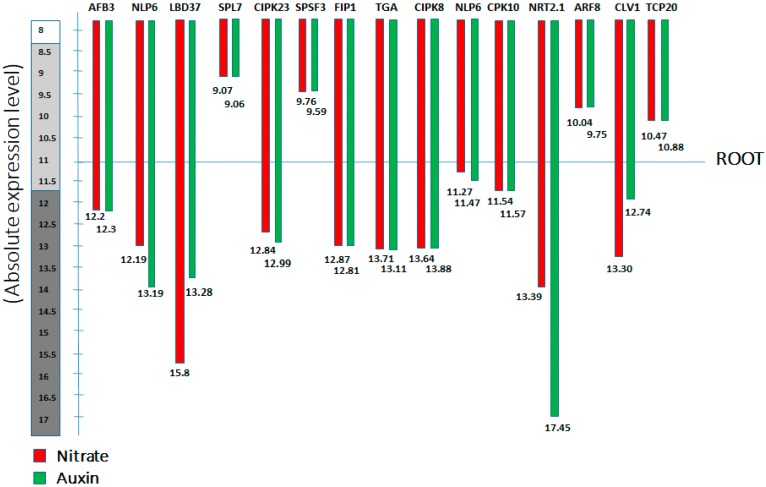
Affymetrix *Arabidopsis* ATH1 genome microarray analysis of the root; The schematic model represents the absolute expression level of the different nitrate transcription factors in the root of *Arabidopsis*, under nitrate (NO_3_^−^) and auxin treatment, the red bar represents the gene expression for nitrate treatment, while the green bar represents the expression level for auxin treatments. (See text for further detail).

Several nitrate responsive genes involved in nitrate signalling promotes lateral root growth. And these genes are characterized in terms of nitrate regulatory genes, which build up signalling overlap between nitrate and auxin, and subsequently induce LR initiation, emergence, and development [50]. We distinguished these genes as short-term and long-term nitrate responsive transcription factors, highlighted their possible roles and also elucidate how they mediate auxin biosynthesis to initiate LRs in *Arabidopsis* at different nitrate levels (Figure 1) (Table 1, Table 2, Table 3 and Table 4). To further demonstrate this signalling crosstalk, here, we have presented the absolute expression by collecting information from “Genevistigator database” (Table S1 and Figure 5), namely the Affymetrix *Arabidopsis* ATH1 genome microarray analysis of root, which further presents each gene’s absolute expression in response to nitrate and auxin treatment respectively. Moreover, a refined set of genes was designated to be regulated by both nitrate (NO_3_^−^) and auxin signalling. We found that these genes have significantly higher levels of baseline expression to NO_3_^−^ and auxin (Figure 5).

Thus, our results demonstrate the identical regulation of these transcription factors for both nitrate and auxin treatments in the root of *Arabidopsis thaliana*. These discoveries lead to new speculation that nitrate plays a vital role in enhancing the effect of auxin signal. Additionally, cis-regulatory elements (CREs) in the promoters of these genes imply their role as candidates that might stimulate the NO_3_^−^ regulated genes expression. This review highlights the ongoing innovation about the several regulatory modules controlling NO_3_^−^ uptake and promoting root growth in response to mutual nitrate and auxin signalling in the model plant *Arabidopsis thaliana*.

However, limited studies assessed the role of auxin in nitrate induced lateral root growth. There has been an outstanding achievement in our understanding of NO_3_^−^ signalling pathways. With the schematic diagram, we have presented the nitrate mediated auxin biosynthesis regulates LRs growth. This regulation is via co-signalling induced by the primary nitrate transporter *NPF6.3/NRT1.1* and its downstream TFs, including *NIA1, NIA2, NiR, NR,* and *NRT2.1* (Figure 2), which mediate IAA biosynthesis induced lateral root growth (Figure 1). However, distinctive transcription factors such as *CBL7, NLP7, TGA1/TGA4, TCP20, NRG2, RSA1, CLV1/CLE1, RSA1, NAC4/OBF4* (downstream of *AFB3*), *SPL9, CLE/CLV1, LBDs, JR1, PHO1, DAAR2* are nitrate responsive, also may target *NPF6.3/NRT1* and *NRT2.1.* However, how these transcription factors interact to build up a signalling module to regulate the LR root growth of *Arabidopsis thaliana* in response to nitrate is still questionable. Furthermore, some nitrate responsive genes are yet to be characterized and their interaction with auxin is yet unknown. However, they are the fundamental contributor genes in the regulation of LR development.

The NO_3_^−^ and IAA signalling overlap in the *Arabidopsis* root system open up a huge research gap for each identified effect on root growth. Hence, the functional identification and characterization of various players associated with NO_3_^−^ signalling pathways and their possible functional interaction with auxin in regulating LRs of *Arabidopsis* is the following step to comprehend the N responses in the plant. Moreover, it is also of much interest to uncover evidence to support the auxin signalling pathways that are self-regulated as they are involved in the feedback regulation of nitrate transport and assimilation to induce lateral root growth and development, which is fundamental to the field of crop biotechnology.

## Figures and Tables

**Table 1:** Short Term Primary Nitrate Responsive Genes.

Gene	Gene Family	Treatment	Transcriptionally NO_3_^−^ Responsive	Effect On RSA	Target Genes	Root Expression Profile	Subcellular Localization	Refs
NLP6	Plant regulator RWP-RK family protein	low	no	support root meristem growth under N starvation	NRT2.1, NRT2.2, and NIA	stele, root hair	nucleus	[23,24]
LBD39,37,38	LBD	high	yes	cell cycle progression of the pericycle	NIA NRT1.1, NRT2.1, NRT2.2, and NRT2.5	root meristem	mitochondrion	[25,26,27]
SPL9	SPL	medium	yes	LR and PR induction	NiR, NIA2, and NRT1.1 (potential)	unknown	cytoplasm /nucleus	[28,29]
NIGT1s	NIGT	low	yes	primary root growth	NRT2.1, *NRT2.4*	expressed in roots (lower panel)	nucleus	[30,31,32]
CIPK23	CBL-interacting protein kinase	low	yes	Primary root growth	Phosphorylating NRT1.1	guard cells and root hairs	cytosole, plastid, chloroplast, cytoplasm, cytosol, nucleus, plasma membrane, plastid	[33,34,35]
NRG2	Putative bZIP	unknown	no	unknown	regulating NRT1.1 and interacting with NLP7	stele, root tip	nucleus	[36]
CPSF30	Polyadenylation specificity factor	unknown	no	PNR/ Nitrate accumulation in roots	alternative polyadenylation	unknown	nucleus	[26]
FIP1	Factor interacting with poly(A) polymerase 1	unknown	no	PNR in the root	polyadenylation of NRT1.1	unknown	nucleus, cytosle, mitochondria, vacule, golgi,	[29]
NRT1.1	NPF	dual	yes	LRs inhibition and stimulation	regulating CIPK8, CIPK23, TGA1/4	root cap and root tip	plasma membrane	[37,38]

**Table 2:** Long Term and Short Term Nitrate Responsive Genes.

Gene.	Gene Family	Treatment	Transcriptionally NO_3_^−^ Responsive	Effect On RSA	Target Genes	Root Expression Profile	Subcellular Localization	Refs
TGA1/4	bZIP	high	yes	LRs induction (initiation)	NRT2.1 and NRT2.2	pericycle and root hair	nucleus	[39]
CIPK8	CBL-interacting	high	yes	Primary root growth	unknown	guard cells and root hairs	cytosol, nucleus	[40,41]
NLP7	RWP-PK Subgroup	low/high	no	LRs and primary root repression	NRT2.1, NiR, NRT2.2, and NIA	stele, root hair, endodermis, root tip	nucleus	[42,41]
CPK10	Ca^2+^-sensor protein kinase	low	yes	Primary root growth	phosphorylate NLP7	unknown	nucleus	[43]

**Table 3:** Long Term Nitrate Responsive Genes.

Gene	Gene Family	Treatment	Transcriptionally NO_3_^−^ Responsive	Effect On RSA	Target Genes	Root Expression Profile	Subcellular Localization	Refs
ANR1	MADS-box	low	yes	LRs induction (elongation)	regulating NRT1.1	LRs (primordia, base and apex), stele	nucleus	[44]
NRT2.1	NPF	high	yes	repressor of LRs initiation	unknown	root cortical and epidermal cells	plastid, nucleus	[45,46,47]
ARF8	Auxin response factors	high	yes	LRs induction (initiation)	unknown regulating	pericycle, root cap stele,	nucleus	[48]
miR167	microRNA	high	yes	LRs induction (initiation)	regulating the expression of ARF8	unknown	nucleus	[43]
miR393	microRNA	high	yes	LRs formation	specifically cleaving AFB3	unknown	nucleus	[42]
AFB3	Auxin receptor	high	unknown	LRs induction and PRs suppression	unknown	unknown	nucleus	[49]
NAC4	NAM/ATAF/CUC	high	yes	LRs induction	OBP4	unknown	nucleus	[49]
CLE	CLAVATA3/ESR-related XI	low	unknown	LRs repression (development and emergence)	CLV1	expressed in the pericycle	extracellular region of the cell	[50]
CLV1	XI LRR-RLKs	low	unknown	LRs repression (development and emergence)	feedback regulation of CLE	expressed in the pericycle	plasma membrane	[51]
CEP	CEP Component	unknown	unknown	Inhibits LRs formation	unknown	LRs primordial and meristem region	extracellular, golgi, endoplasmic reticulum	[52]
HIN9/IWS1	Component of RNAPII complexes	high	no	regulation of root NO_3_^–^ uptake	NRT2.1	unknown	nucleus	[53]
TCP20	TCP	low	no	LRs development	NRT1.1, NIA, NRT2.1, and NiR Roles	stele, root cap	nucleus	[54]

**Table 4:** Unknown Affinity Nitrate Responsive Genes.

Gene	Gene Family	Treatment	Transcriptionally NO_3_^−^ Responsive	Effect On RSA	Target Genes	Root Expression Profile	Subcellular Localization	Refs
TAR2	Tryptophan Aminotransferase Related 2	low	unknown	maintenance of the root stem cell niches	unknown	root meristem and root vascular system	endoplasmic reticulum membrane, integral component of membrane	[36]
JR1	Mannose-binding lectin superfamily protein	low	unknown	root allometry	unknown	root	nucleolus, vacuole, etc.	[58]
DAAR2	Phenazine biosynthesis PhzC/PhzF protein	low	unknown	LR induction	unknown	root	cytosol, nucleus	[58]
RSA	ATP/GTP-binding protein family	high	unknown	LRs induction (elongation)	unknown	root	cytoplasm, cytosol, nucleus	[82]
PHO1	Phosphate 1	high	unknown	root allometry	unknown	root	golgi apparatus, golgi membrane, cytoplasm,	[82]

## Supplementary Materials

The following are available online at https://www.mdpi.com/1422-0067/21/8/2880/s1, Table S1. Affymetrix Arabidopsis ATH1 Genome Microarray of Anatomical Part: Root.

## Abbreviation

RSA	Root system architecture
LBD	Lateral Organ Boundaries Domain
SPL	SQUAMOSA PROMOTER BINDING PROTEIN-LIKE
NIGT1	NITRATE-INDUCABLE GRAP-TYPE TRANSCRIPTIONAL REPRESSOR1
FIP1	Factor interacting with poly (A) polymerase 1
ARF	Auxin Response Factor
HNI9	HIGH NITROGEN INSENSITIVE9
NLP	NIN-like protein
TCP20	The branched1/cycloidea/proliferating cell factor1-20
XTH	Xyloglucan endotransglucosylases
JR1	JOSMONATE RESPONSIVE 1
DAAR2	D AMINO ACID RACEMASE2
HNI9/AtIWS1	HIGH NITROGEN INSENSITIVE9
PHO1	PHOSPAHT TRANSPORTER 1
RSA1	ROOT SYSTEM ARCHITECTURE 1
NRG2	NITRATE REGULATORY GENE 2
TIR1/AFB	TRANSPORT INHIBITOR RESPONSE1/AUXIN SIGNALLING F-BOX
AUX/IAA	AUXIN/ INDLOE-3-ACETIC ACID
ARF	AUXIN RESPONSE FACTOR
CPSF30-L	cleavage and polyadenylation specificity factor 30-L
SCFTIR/AFB	S-PHASE KINASE ASSOCIATED PROTEIN 1-CULLIN-F-BOX PROTEIN (SCF)-type E3 ubiquitin-protein ligases
MeJA	Methyl jasmonate
EMSA	Electrophoretic mobility shift analysis
