# Supplementary material for: Signalling Overlaps between Nitrate and Auxin in Regulation of The Root System Architecture: Insights from the Arabidopsis thaliana
Source: Int J Mol Sci. 2020 Apr 20;21(8):2880. doi: 10.3390/ijms21082880 (PMC7215989; doi:10.3390/ijms21082880)
Supplement: Supplementary file 1 [file ijms-21-02880-s001.pdf]

Table S1. Affymetrix Arabidopsis ATH1 Genome Microarray of Anatomical Part: Root

Gene	Detailed view of expression level			Anatomical part	Developmental stages	Stimulus	genotypes	Global sample characteristic
	Sample name	Original ID	Experimental					
AFB3	Auxin 8_wt_IAA_ 3h_roo_2-3	GSM871271	AT-00660	Root	Cotyledon fully open (1.00)	IAA	Col-0	wild type
	Auxin 8_wt_IAA_ 3h_roo_2-3	GSM469820	AT-00388	Root	Seedling 1.02	KNO3	Col-1	wild type
NLP7	Auxin 7_wt_IAA_24h_ 16-2	GSM1030589	AT-00655	Root	1.00	IAA	Col-0	wild type
	Nitrate res. 3_wt_KNO3_20 min_13-2	GSM501209	AT-00434	Root	1.03	KNO3	Col-0	Wild type
LBD37	Nitrate res. 4_afb3- 1_KNO3_2h_4-3	GSM870362	AT-00610	Root	1.05	KNO3	afb3-1	Non-Wild type
	Auxin 7_wt_IAA_1h_1 1-2	GSM1030600	AT-00655	Root	1.00	IAA	Col-0	Wild type
SPL9	Auxin 7_wt_IAA_1h_1 1-1	GSM1030568	AT-00655	Root	1.00	IAA	Col-0	Wild type
	Nutrient 8_wt_nitr. 0mM_suc. 30mM_2-1	LL33-17	AT-00209	Root	6.30	NO3	Col-0	Wildtype

CIP23	Auxin 8_wt_IAA_3h_roo_2-2	GSM871270	AT-00660	Root	1.00	IAA	Col-0	Wild type
	Nutrient 8_wt_nitr. 15mM_suc. 0mM_10-2	LL33-16	AT-00209	Root	6.30	Nitrate	Col-0	Wild type
SPSF30	Auxin 7_wt_IAA_12h_15-2	GSM1030588	AT-00655	Root	1.00	IAA	Col-0	Wild type
	Nitrate res._chl1_NO3_4-2	GSM231203	AT-00266	Root	1.01	Nitrate	CHL1-5	Non-wild types
FIP1	Auxin 7_wt_IAA_0.5h_10-3	GSM1030599	AT-00655	Root	1.00	IAA	Col-0	Wild type
	Nutrient 8_wt_nitr. 5mM_suc. 0mM_7-1	LL34-2	AT-00209	Root	6.30	Nitrate	Col-0	Wild type
TGA1	Auxin 7_wt_IAA_24h_16-3	GSM1030605	AT-00655	Root	1.00	IAA	Col-0	Wild type
	Nutrient 8_wt_nitr. 0mM_suc. 90mM_4-1	LL33-19	AT-00209	Root	6.30	Nitrate	Col-0	Wild type
CIPK8	Auxin 8_wt_IAA_3h_roo_2-3	GSM871271	AT-00660	Root	1.00	IAA	Col-0	Wild type

	Nitrate res._wt_NO3_3-2	GSM231197	AT-00266	Root	1.01	Nitrate	Col-0	Wild type
NLP6	Auxin 7_wt_IAA_0.5h_10-2	GSM1030583	AT-00655	Root	1.00	IAA	Col-0	Wild type
	Nitrate res._chl1_NO3_4-1	GSM231202	AT-00266	Root	1.01	Nitrate	CHL1-5	Non-wildtype
CPK10	Auxin 7_wt_IAA_8h_14-2	GSM1030587	AT-00655	Root	1.00	IAA	Col-0	Wild type
	Nitrate res._wt_NO3_3-3	GSM231198	AT-00266	Root	1.01	Nitrate	Col-0	Wild type
ANR1	Auxin 8_wt_IAA_3h_roo_2-2	GSM871270	AT-00660	Root	1.00	IAA	Col-0	Wild type
	Nutrient 8_wt_nitr. 0mM_suc. 30mM_2-1	LL33-17	AT-00209	Root	1.00	Nitrate	Col-0	Wild type
NRT2.1	Auxin 7_wt_IAA_4h_13-1	GSM1030570	AT-00655	Root	1.00	IAA	Col-0	Wildtype
	Nutrient 8_wt_nitr. 0mM_suc. 30mM_2-1	LL33-17	AT-00209	Root	6.30	Nitrate	Col-0	Wild type

ARF8	Auxin 7_wt_IAA_24h_16-2	GSM1030589	AT-00655	Root	1.00	IAA	Col-0	Wildtype
	Nitrate res._chl1_NO3_4-2	GSM431203	AT-00266	Root	1.01	Nitrate	Col-0	Wild type
CLV1	Auxin 7_wt_IAA_12h_15-3	GSM1030604	AT-00655	Root	1.00	IAA	Col-0	Wild type
	Nutrient 8_wt_nitr. 0mM_suc. 30mM_2-2	LL34-5	AT-00209	Root	6.30	Nitrate	Col-0	Wildtype
TCP20	Auxin 7_wt_IAA_2h_12-1	GSM1030569_	AT-00655	Root	1.00	IAA	Col-0	Wild type
	Nutrient 8_wt_nitr. 0mM_suc. 30mM_2-2	LL34-5	AT-00209	Root	6.30	Nitrate	Col-0	Wildtype
